# On the Effects of Core Microstructure on Energy Absorbing Capabilities of Sandwich Panels Intended for Additive Manufacturing

**DOI:** 10.3390/ma15041291

**Published:** 2022-02-09

**Authors:** Valerio Acanfora, Rossana Castaldo, Aniello Riccio

**Affiliations:** Department of Engineering, University of Campania “L. Vanvitelli”, via Roma, 29, 81031 Aversa, Italy; rossana.castaldo@studenti.unicampania.it (R.C.); aniello.riccio@unicampania.it (A.R.)

**Keywords:** crashworthiness, composite materials, additive manufacturing, low-velocity impact simulations, FE model

## Abstract

Increasing transportation safety can be observed as one of the biggest engineering challenges. This challenge often needs to be combined with the need to deliver engineering solutions that are able to lower the environmental impact of transportation, by reducing fuel consumption. Consequentially, these topics have attracted considerable research efforts. The present work aims to address the previously cited challenges by maximizing the energy absorption capabilities of hybrid aluminum/composite shock absorbers with minimal thickness and mass. This engineering solution makes it possible to lighten vehicles and reduce fuel consumption, without compromising safety, in terms of crashworthiness capabilities. A numerical sensitivity study is presented, where the absorbed energy/mass (AE/m) and the absorbed energy/total panel thickness (AE/H_tot_) ratios, as a consequence of low-velocity impact simulations performed on six different shock absorbers, are compared. These hybrid shock absorbers have been numerically designed by modifying the core thickness of two basic absorbers’ configurations, characterized, respectively, by a metallic lattice core, intended to be produced through additive manufacturing, and a standard metallic honeycomb core. This work provides interesting information for the development of shock absorbers, which should be further developed with an experimental approach. Indeed, it demonstrates that, by integrating composite skins with a very light core producible, by means of additive manufacturing capabilities, it is possible to design shock absorbers with excellent performance, even for very thin configurations with 6 mm thickness, and to provide a significant increase in AE/m ratios when compared to the respective equal volume standard honeycomb core configurations. This difference between the AE/m ratios of configurations with different core designs increases with the growth in volume. In detail, for configurations with a total thickness of 6 mm, the AE/m increases in additive manufacturing configurations by approximately 93%; for those with a total thickness of 10 mm, the increase is 175%, and, finally, for those with a total thickness of 14 mm, the increase is 220%.

## 1. Introduction

The development of structural engineering solutions that are able to ensure safety in transportation is an aspect of structural design that is as old as the idea of transportation itself.

Among the different solutions, shock absorbers have been found to be the best compromise between performances, in terms of crashworthiness and weight. However, the energy absorption capabilities are influenced by different design parameters, such as the shock absorber’s thickness, materials, and core microstructures. Hence, investigations are mandatory to assess the influence of variation in the parameters of the shock absorber, in terms of its crashworthiness capability. 

The first shock absorbers [1,2] had the exclusive task of smoothing out the rolling and pitching caused by the motion of vehicles on rough roads.

It was only with the increase in speed in transport systems that the need to install devices as structural components to improve the passive safety of drivers and passengers in the case of accidents emerged. In the aviation field, this topic was addressed with considerable prominence, as early as the middle of the 20th century, by the pilot Hugh DeHaven. After surviving an aircraft accident, he spent considerable effort producing a guideline for the design of integrable shock absorbers in key aircraft positions [3,4]. Nowadays, sandwich structures are among the best-performing cushioning solutions. In particular, they can be categorized as metallic, polymeric, and hybrid sandwich structures.

The first category of sandwich structures, being characterized by both a metallic internal core and skins, allows the absorption of high rates of impact energy thanks to the extraordinary plasticization capabilities of metals. However, absorbers belonging to this category are almost always heavy [5,6,7].

The second category of sandwich structures, on the other hand, is characterized by an internal core and skins, both made of plastic materials, which are usually lighter when compared to metallic materials. However, their energy absorption capability, related to elastic and fracture mechanisms, is far less than that provided by metallic absorbers [8,9].

Therefore, a good trade-off may be possible thanks to hybrid sandwich structures. Indeed, by combining the plasticising capabilities of a metal core with the lightness of a polymer coating, often stiffened with composite materials, they can reach high absorbed energy/mass (AE/m) and absorbed energy/total panel thickness (AE/H_tot_) ratios [10,11,12].

The above-mentioned works help to contextualize the advantages provided by hybrid sandwich absorbers. In Ref. [10], the vacuum-assisted resin transfer moulding (VARTM) processing technique was used to produce composite sandwich plates with composite laminate facesheets combined with an aluminium foam core [10]. These plates are thick, at least 16 mm, but they are able to absorb a high proportion of the energy produced during low-speed impact at around 15 J. The work presented in Ref. [11] investigates the impact resistance and residual performance of metal foam-based sandwich panels [11]. In particular, a series of experimental impact tests on sandwiches with an aluminium foam core, and skin manufactured with different geometry and composite material systems, was performed, in order to provide guidelines for the design of hybrid sandwich structures for automotive-related applications. Another application of hybrid sandwich shock absorbers is presented in Ref. [12], where the mitigation capabilities of auxetic honeycomb sandwich panels against shock loads caused by explosive events are explored, both numerically and experimentally [12].

Hence, these hybrid structures seem to be compliant with the transportation industry’s need to reduce the mass of structural components and, consequently, fuel consumption [13,14,15,16,17].

As demonstrated in the previous articles, in order to ensure affordable energy absorption capabilities, these devices must be at least more than 16 mm thick. Therefore, the development of solutions that are able to provide excellent energy absorption characteristics, with a reduced thickness and mass correlate with the adoption of innovative manufacturing techniques, such as the additive manufacturing technique.

According to this enabling technology, complex geometries can be 3D printed in both polymer and metallic materials [18,19,20,21]. In particular, with L-PBF metal additive technologies, due to the micron precision of the sintering laser, it is possible to achieve ad hoc lattice domain maximising energy performance, while retaining internal voids and limited thickness. Details on the capabilities of the metal additive printing process are available in Ref. [20], while information about the shock absorption and toughness, as a representative factor for the energy absorption of BCC and FCC materials, is provided in [21].

Therefore, this technique may lead to the development of new, compact and extraordinarily efficient shock absorption concepts. This evaluation is the research topic of the authors of this paper. In particular, they explored the aspects already covered in Ref. [22]. 

In this paper [22], the energy absorption and mass performance of sandwich panel configurations, at a constant volume, characterized by cores achievable by both common processing techniques (such as CNC) and additive manufacturing (DfAM lattice core), are cross-compared. The work in [22] provides a starting point for the current paper, proved by means of comparisons with numerical and experimental study data from the literature, stating that sandwich structures with cores topologically optimised for additive manufacturing exhibit higher performance, in terms of energy absorption, than sandwich structures with a standard core configuration. 

This innovative concept of shock absorbers designed for additive manufacturing, presented in [22], has been adopted as a starting point for the present work. In particular, this work reports a numerical comparison of the effectiveness, measured in terms of absorbed energy/mass and absorbed energy/thickness ratios between two different hybrid shock absorber concepts, using FE models already validated in [22], in order to provide useful design considerations for the development of a shock absorber that maximises energy absorption capabilities with a minimum possible mass and volume footprint.

The first hybrid shock absorber concept is characterised by an aluminium honeycomb core that can be fabricated using standard CNC machining techniques (cutting to size, drilling, and forming); whereas, the second hybrid shock absorber concept has a metal BCC lattice core, based on a unit cell microstructure already optimised for additive manufacturing in [22]. Among the unit cell configurations examined in [22], the BCC configuration cell was chosen for the development of the numerical models presented in this paper, because the results of sandwich panels with core unit cell configurations were thoroughly compared with experimental and numerical data from the literature. These comparisons are presented in [22].

Therefore, by setting the core thickness to three different levels (4 mm, 8 mm, and 12 mm), the structural responses of six configurations were compared, three with cores designed for additive manufacturing and three with honeycomb cores.

The second section of the article describes the developed numerical models, focusing on the applied boundary conditions in the frame of the performed simulations. Finally, in Section 3, the results obtained for the different configurations are presented, compared and commented upon.

## 2. Materials and Methods

To quantify the effective contribution provided by the additive technologies, this work compares the energy performance, in terms of absorbed energy/mass and absorbed energy/volume ratios, by performing numerical low-velocity impact simulations at 20 J on hybrid (composite metallic) shock absorbers. Hybrid shock absorbers with a common honeycomb structure were compared with hybrid shock absorbers with a lattice core manufacturable exclusively by metal additive printing technology.

**Figure 1:**
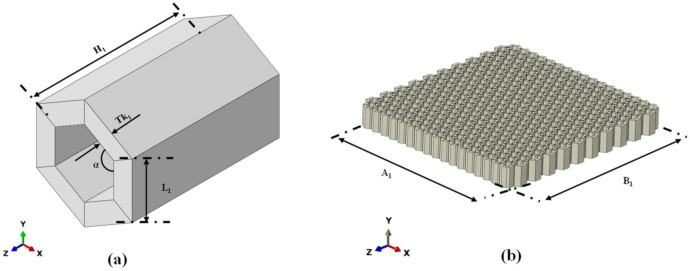
Honeycomb core: (**a**) unit cell; (**b**) cell distribution in core.

**Figure 2:**
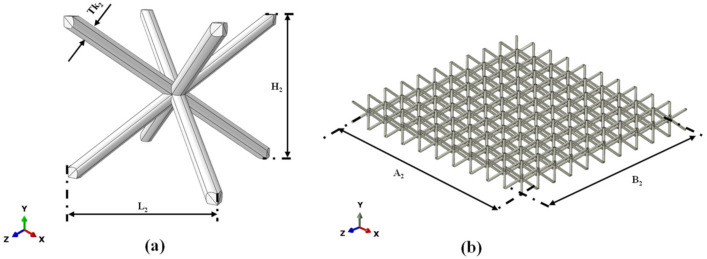
BCC core: (**a**) unit cell; (**b**) cell distribution in core.

Figure 1a,b show, respectively, the hexagonal unit cell of the honeycomb core in an isometric view and its distribution in the sandwich panel, while Figure 2a,b show, respectively, the BCC unit cell of the lattice core and its distribution in the sandwich panel. 

**Figure 3:**
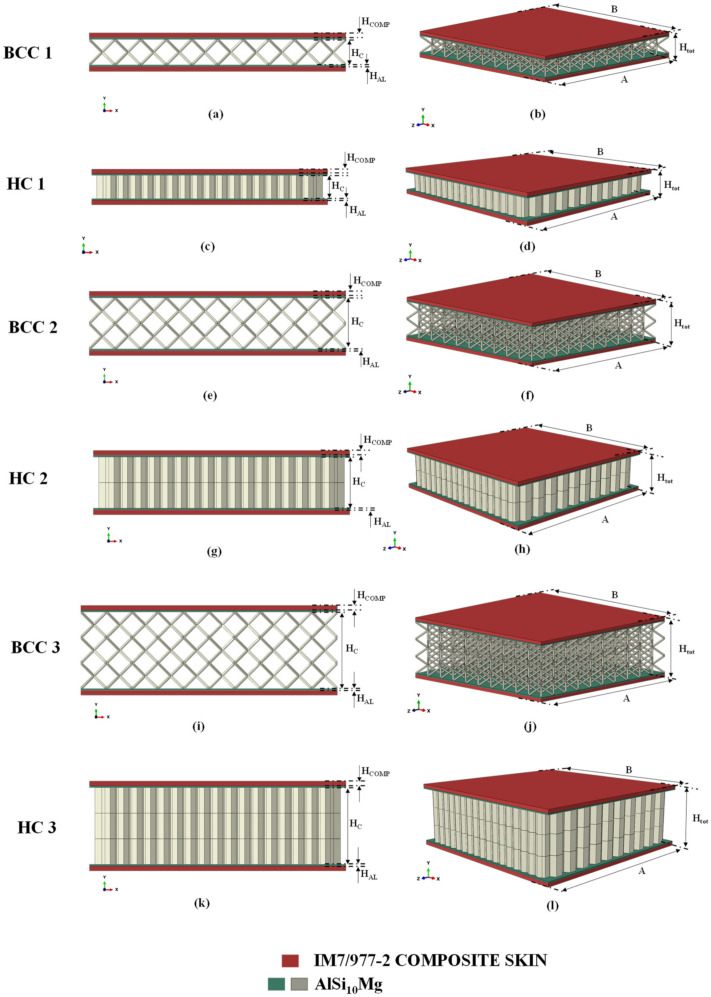
Hybrid shock absorbers with different cores: (**a**,**b**) BCC 1; (**c**,**d**) HC 1; (**e**,**f**) BCC 2; (**g**,**h**) HC 2; (**i**,**j**) BCC 3; (**k**,**l**) HC 3.

By varying the H_c_ parameter, defined in Figure 3, the height of the sandwich structure has been changed; hence, a total of six sandwich absorber configurations have been generated. Indeed, three configurations, 4 mm, 8 mm, and 12 mm thick, for the honeycomb core type, named, respectively, HC1, HC2, and HC3, have been defined. Similarly, three configurations, 4 mm, 8 mm, and 12 mm thick, for the honeycomb lattice type, named, respectively, BCC1, BCC2, and BCC3, have been identified, as shown in Figure 3.

Figure 3 also shows that each sandwich structure is composed of an internal aluminium alloy core, an internal aluminium skin, with thickness H_AL,_ and an external CFRP (carbon fibre-reinforced polymer) skin composed of four plies, each one with a thickness of 0.1875 mm and oriented according to the stacking sequence (0; −45; 45; 90).

The integration of the composite material on the metallic structure was designed to increase the stiffness/mass and strength/mass ratios of the analysed configurations, and, at the same time, as demonstrated in [22], allow a gradual distribution, over time, of the accelerations arising during the impact phenomenon.

Table 1 lists the geometrical parameters characterising the unit cells and the sandwich structures.

The properties of the considered material systems are reported in Table 2 and Table 3, respectively, for the elastic and plastic properties of the AlSi_10_Mg powder [23], and in Table 4 for each IM7/977-2 ply.

In order to accurately simulate the true physical behaviour of panels by numerical simulation in the Abaqus finite element code, the 3D elements have been adopted.

In particular, the core, the impactor and the metal skins were discretized using C3D8R elements, while SC8R continuum shells were chosen for the composite coating.

An element size of 0.75 mm was chosen according to a mesh sensitivity impact analysis at 6 J on the BCC1 configuration, performed in [22]. Therefore, the reliability of the adopted numerical models is guaranteed by the numerical–experimental comparisons presented in [22]. 

**Figure 4:**
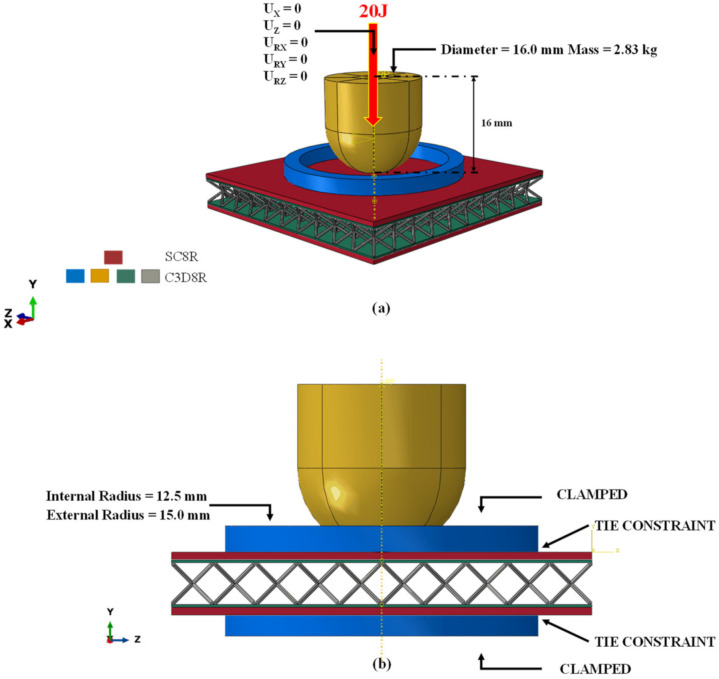
Definition of mesh elements (**a**) and applied boundary conditions (**b**).

In Figure 4, the model parts are shown with emphasis on the adopted finite element formulation. This figure also shows the boundary conditions, applied to the sandwich structures, chosen to simulate the low-velocity impact event according to the ASTM D7136 standard.

In detail:

- The impactor and rings were considered as rigid bodies;
- The impactor was constrained to move only along the vertical Y axis, while the rings were clamped;
- To reproduce an impact with an energy level of 20 J, a 2.83 kg impactor has been moved along Y at a speed of 3759.56 mm/s;
- The contact between the outer surfaces of the sandwich and the rings was ensured by means of tie constraints;
- A tangential friction coefficient of 0.3 was considered;
- The connection between the metal and composite plies was set using tie constraints;
- The connection between the core and the metal ply has been defined through tie constraints.

## 3. Results

In this chapter, the results of the explicit FEM simulations of the structural response of the analysed hybrid shock absorbers, as a consequence of an impact of 20 J, are presented.

**Figure 5:**
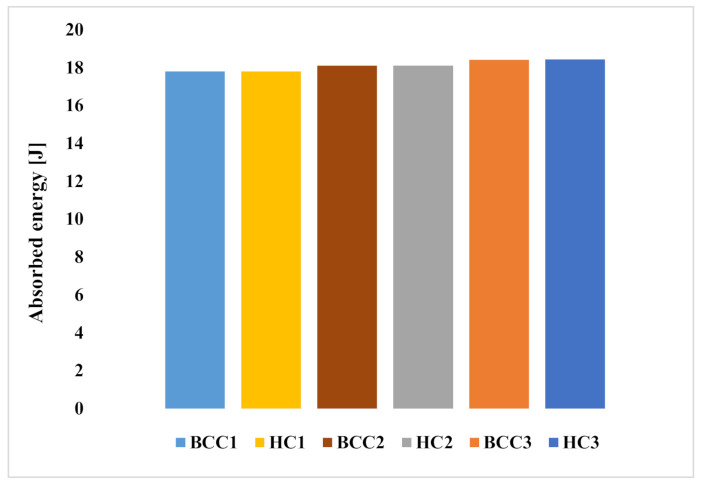
A comparison between the absorbed energies of the examined configurations after a 20 J numerical impact test.

Figure 5 compares the absorbed energies at the end of the numerical impact simulation. This absorbed energy has been evaluated by calculating the sum of the plastic, damage, and viscous energy rates when the impact phenomenon stabilizes.

As can be observed from the histogram chart, all the analysed configurations exhibit an excellent energy absorption capability, as they all absorb more than 89% of the impact energy. This energy absorption percentage of lattice core sandwich structures is in line with the results of Vrana et al., in [24]. In detail, the amount of energy absorbed by the configurations is 17.8 J for the BCC1 and HC1 configurations, 18.1 J for the BCC2 and HC2 configurations, 18.41 J for the BCC3 configuration, and 18.43 J for the HC3 configuration.

Hence, increasing the sub-unit size of the lattice core structure results in a very slight increase in the energy absorption capabilities. 

Figure 5 also shows how, with the same H_c_, structures with cores lightened by means of additive manufacturing achieve energy absorption performances that are practically identical to honeycomb cores with corresponding configurations, thus achieving a higher AE/m ratio. 

In detail, the calculated AE/m ratios are as follows:

- 1407.35 J/kg for the BCC1 configuration;
- 729.51 J/kg for the HC1 configuration;
- 1263.75 J/kg for the BCC2 configuration;
- 460.11 J/kg for the HC2 configuration;
- 1150.41 J/kg for the BCC3 configuration;
- 359.68 J/kg for the HC3 configuration.

**Figure 6:**
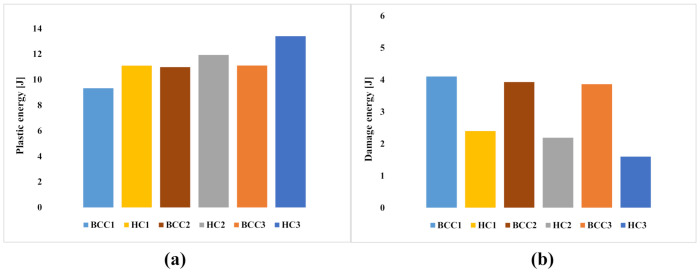
Energy absorbed in plastic deformation (**a**) and in damage (**b**).

By comparing the plastic (Figure 6a) and damage (Figure 6b) contributions to energy absorption, it is possible to assess the mechanisms according to which the energy generated during the impact phenomenon was absorbed by the investigated structures. From Figure 6a, it can be observed that an increase in core thickness H_c_ leads to an increase in the plastic contribution, due to the increase in the metallic part in the hybrid structures. Consequently, the rate of energy absorbed in damage decreases when increasing H_c._

Finally, the comparison in Figure 6b also shows that, in the configurations with a BCC cell, the composite covering assumes a more decisive role. Indeed, at the same thickness, the rate of energy absorbed in damage by the structure with the additive core is significantly higher than in the structure with a standard honeycomb core. The energy absorbed in damage mechanisms is insensitive to the increase in thickness. 

**Figure 7:**
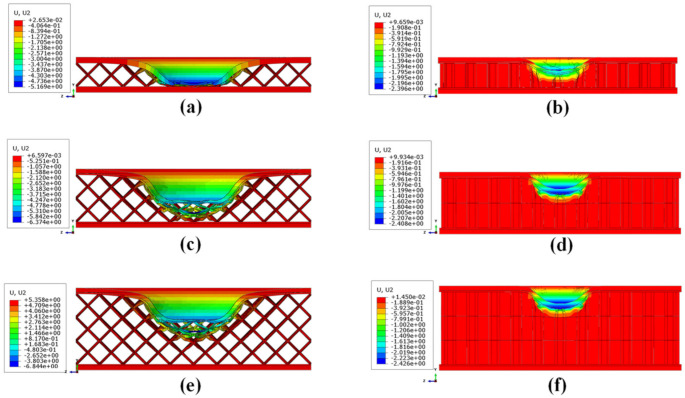
Out-of-plane displacements of the investigated structures: (**a**) BCC1; (**b**) HC1; (**c**) BCC2; (**d**) HC2; (**e**) BCC3; (**f**) HC3.

The trend of the energy absorbed by plastic deformations is also confirmed by the out-of-plane plastic deformations at the end of the simulation, shown in Figure 7.

As can be expected, the indentation is greater in the maximum thickness configurations. In particular, it reaches a maximum value of 6.84 mm in the configuration with the 12 mm BCC core.

**Figure 8:**
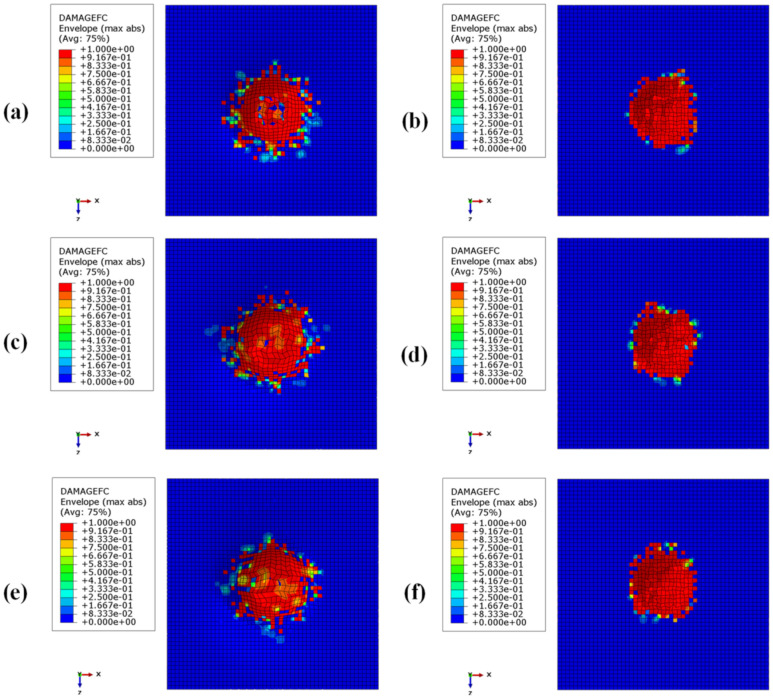
Intralaminar damage recorded in the upper composite coating, evaluated according to the Hashin criterion. Fibre fracture for compressive loads: (**a**) BCC1; (**b**) HC1; (**c**) BCC2; (**d**) HC2; (**e**) BCC3; (**f**) HC3.

**Figure 9:**
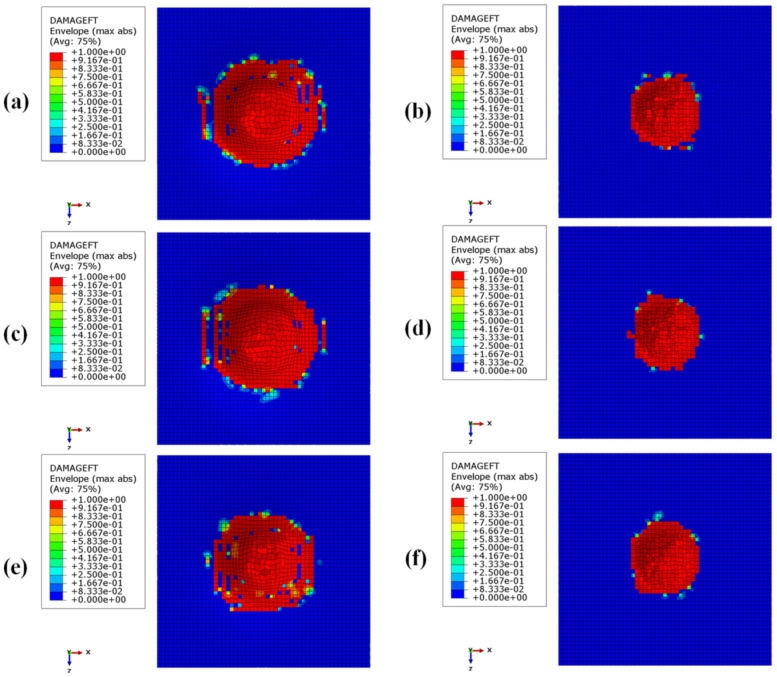
Intra-laminar damage recorded in the upper composite coating, evaluated according to the Hashin criterion. Fibre fracture for tensile loads: (**a**) BCC1; (**b**) HC1; (**c**) BCC2; (**d**) HC2; (**e**) BCC3; (**f**) HC3.

**Figure 10:**
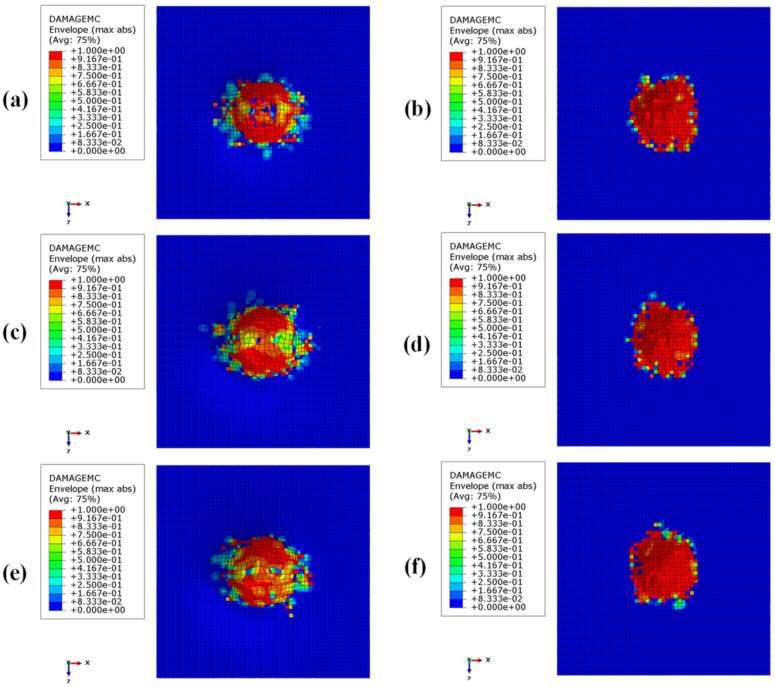
Intra-laminar damage recorded in the upper composite coating, evaluated according to the Hashin criterion. Matrix fracture for compressive loads: (**a**) BCC1; (**b**) HC1; (**c**) BCC2; (**d**) HC2; (**e**) BCC3; (**f**) HC3.

**Figure 11:**
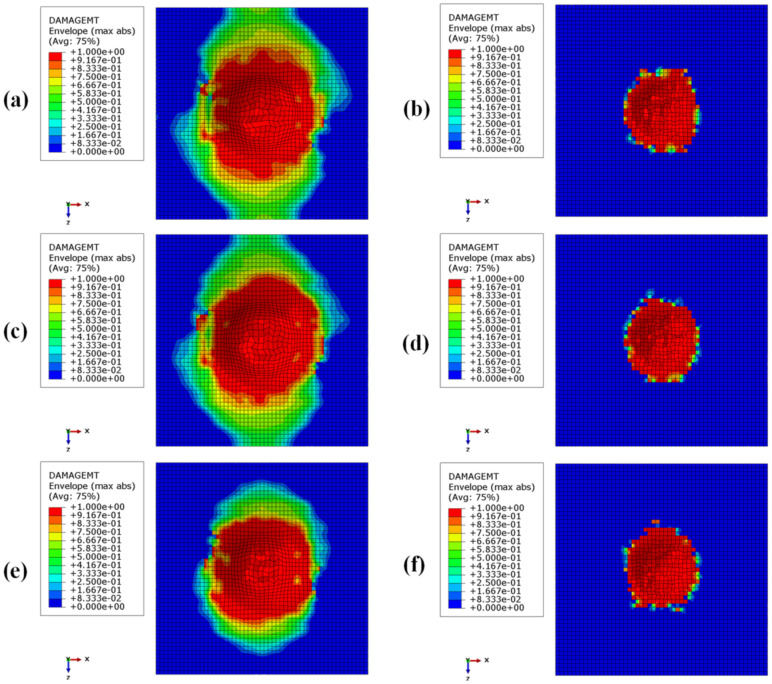
Intra-laminar damage recorded in the upper composite coating, evaluated according to the Hashin criterion. Matrix fracture for tensile loads: (**a**) BCC1; (**b**) HC1; (**c**) BCC2; (**d**) HC2; (**e**) BCC3; (**f**) HC3.

Figure 8, Figure 9, Figure 10 and Figure 11 show the envelope of intralaminar damages, evaluated according to the Hashin criteria, in the four upper layers of the panels. In particular, Figure 8 shows the compressive failure of the fibres, Figure 9 shows the tensile damage of the fibres, Figure 10 shows the compressive damage of the matrix, and Figure 11 shows the tensile damage of the matrix. 

Comparing these figures, as already remarked when comparing energies, the damaged composite areas are significantly larger in the configurations with a BCC core structure.

This is because, due to the presence of larger void volumes in the lattice core, when compared to the honeycomb core, the impactor can induce larger deformations to the upper skins, producing a more extended damaged area in the composite laminae.

Furthermore, as a result of the decrease in the metallic part, with the decrease in core height (with a consequent decrease in the impact energy absorbed by plastic deformations), the damaged area in the composites is more pronounced in the BCC1 and HC1 configurations. 

In particular, in the BCC1 configuration, such damage also extends into the lower composite skin.

**Figure 12:**
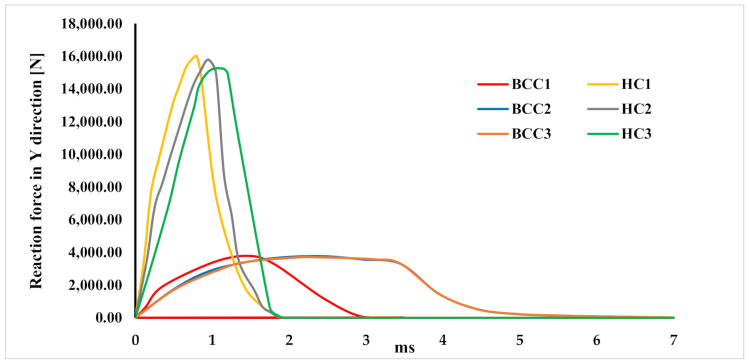
Force-time graph.

Figure 12 compares the force–time curves between the different analysed configurations. It can be observed that the honeycomb configurations exhibit worse behaviour, in terms of crashworthiness, than the BCC configurations. Indeed, as they are more rigid, the honeycomb solutions do not allow gradual dissipation of the acceleration over time. 

In the BCC configurations, the distribution of loads over time is particularly advantageous in terms of crashworthiness capabilities.

This favourable trend is related to the presence of composite skins. Indeed, due to the onset and evolution of failures in composites, the absorber is able to gradually dissipate the energy produced during the impact event.

In order to allow a direct comparison between the tested configurations, Table 5 expresses, as a percentage, the differences, in terms of absorbed energy, between the analysed hybrid absorbers. Finally, in order to select the best configuration, maximizing the energy absorption capabilities, while ensuring the lowest mass and footprint, AE/m and AE/H_tot_ ratios have been introduced. 

It should be noted that, thanks to the lattice configuration of the core, only manufacturable by means of additive technology, the AE/m ratios assume values one order of magnitude higher than those exhibited by the honeycomb core configurations. This is because the cores produced by additive manufacturing are characterized by a very light structure when compared to the honeycomb structure. Furthermore, from the comparison in terms of the AE/H_tot_ ratio, it can be remarked that the configurations that best maximize the energy absorption capabilities are, for both the core configurations, those with the minimum thickness (6 mm).

In conclusion, the presented sensitivity analysis confirms that, among the examined configurations, the configuration that is most efficient in terms of the AE/m and AE/H_tot_ ratios, and that best distributes the loads arising during an impact event, is the BCC1 configuration (i.e., the configuration with the lattice core and minimum thickness). 

## 4. Conclusions

This work assesses the effectiveness of hybrid metal composite shock absorbers, characterised by a lattice core structure, intended to be produced through a metal additive manufacturing process, and compares their energy absorption capabilities with hybrid shock absorbers and with standard honeycomb core configurations. 

In particular, by comparing the AE/m and AE/H_tot_ ratios, it has been demonstrated that configurations with additive manufactured lattice cores are able to maximize the energy absorption capabilities, while preserving thickness and mass reduction.

This is easily deducible from Table 5, which contains a compact comparison of the achieved numerical results. In particular, it is evident that the parameter AE/m, which is the most representative of structural effectiveness, is higher in the lattice than in the honeycomb structures. This gap increases with the total panel thickness. Specifically, the percentage variation in AE/m between the DfAM and standard core configurations is +93% when the total panel thickness is 6 mm, +175% when it is 10 mm, and +220% when it is 14 mm.

## Figures and Tables

**Table 1:** Geometric parameters.

Parameter	BCC 1	HC 1	BCC 2	HC 2	BCC 3	HC 3
A [mm]	40.00	40.00	40.00	40.00	40.00	40.00
B [mm]	40.00	40.00	40.00	40.00	40.00	40.00
A_1_ [mm]	40.00	40.00	40.00	40.00	40.00	40.00
B_1_ [mm]	40.00	40.00	40.00	40.00	40.00	40.00
H_tot_ [mm]	6.00	6.00	10.00	10.00	14.00	4.00
H_C_ [mm]	4.00	4.00	8.00	8.00	12.00	12.00
H_COMP_ [mm]	0.75	0.75	0.75	0.75	0.75	0.75
H_AL_ [mm]	0.25	0.25	0.25	0.25	0.25	0.25
L_1_ [mm]	/	1.50	/	1.50	/	1.50
L_2_ [mm]	4.00	/	4.00	/	4.00	/
H_1_ [mm]	/	4.00	/	8.00	/	12.00
H_2_ [mm]	4.00	/	4.00	/	4.00	/
Tk_1_ [mm]	/	0.5	/	0.5	/	0.5
Tk_2_ [mm]	0.4	/	0.4	/	0.4	/
Mass [kg]	0.06	0.12	0.07	0.19	0.08	0.26

**Table 2:** Elastic and fracture properties of AlSi_10_Mg.

Property	Value
Young’s modulus [GPa]	68
Density [kg/m^3^]	2650
Strain rate [s^−1^]	100–150
Fracture strain for ductile damage	0.065
Stress triaxiality	0.33
Fracture energy [kJ/m^2^]	67

**Table 3:** Plastic properties of AlSi_10_Mg.

Yield Stress [MPa]	Plastic Strain
153	0
160	0.0004
178	0.002
203	0.013
214	0.020
224	0.030
231	0.040
234	0.050
235	0.056

**Table 4:** Mechanical properties of IM7/977-2 composite material.

IM7/977-2 Composite Properties	Value
Density [t/mm^3^]	1.58 × 10^-9^
E1 [GPa]	153.05
E2 = E3 [GPa]	10.30
G12 = G13 [GPa]	6.0
G23 [GPa]	3.7
ν12 = ν13	0.30
ν23	0.40
Longitudinal Tensile Strength [GPa]	1.250
Longitudinal Compressive Strength [GPa]	0.850
Transverse Tensile Strength [GPa]	0.065
Transverse Compressive Strength [GPa]	0.2
Longitudinal Shear Strength [GPa]	0.075
Transverse Shear Strength [GPa]	0.035
Longitudinal Tensile Fracture Energy [kJ/m^2^]	15
Longitudinal Compressive Fracture Energy [kJ/m^2^]	7
Transverse Tensile Fracture Energy [kJ/m^2^]	0.5
Transverse Compressive Fracture Energy [kJ/m^2^]	4

**Table 5:** Comparison of the key parameters used to characterize the effectiveness of the solutions proposed.

Parameter	BCC 1	HC 1	BCC 2	HC 2	BCC 3	HC 3
Total energy [J]	20.00	20.00	20.00	20.00	20.00	20.00
Absorbed energy [%]	88.94	89.01	90.48	87.00	92.03	92.15
Plastic energy [%]	46.67	55.50	54.92	59.67	55.52	67.03
Damage energy [%]	20.5	12.02	19.64	10.95	19.32	8.12
AE/m [J/kg]	1407.35	729.51	1263.75	460.11	1150.41	359.68
AE/H_tot_ [J/mm]	14.82	14.83	9.04	8.70	6.57	6.58

## Data Availability

Not applicable.
